# Threshold of hyperglycaemia associated with mortality in critically ill patients: a multicentre, prospective, observational study using continuous glucose monitoring

**DOI:** 10.1007/s00125-024-06136-1

**Published:** 2024-04-03

**Authors:** Yaxin Wang, Siwan Li, Jingyi Lu, Kaixuan Feng, Xiaoli Huang, Fangbao Hu, Menghan Sun, Yan Zou, Yingchuan Li, Weifeng Huang, Jian Zhou

**Affiliations:** 1 Department of Endocrinology and Metabolism, Shanghai Sixth People’s Hospital Affiliated to Shanghai Jiao Tong University School of Medicine; Shanghai Clinical Center for Diabetes; Shanghai Diabetes Institute; Shanghai Key Laboratory of Diabetes Mellitus, Shanghai, China; 2 Department of Anesthesiology, Tongji University Affiliated Shanghai Tenth People’s Hospital, Shanghai, China; 3 Department of Critical Care Medicine, Shanghai Sixth People’s Hospital Affiliated to Shanghai Jiao Tong University School of Medicine, Shanghai, China; 4 Department of Critical Care Medicine, Jinshan Branch of Shanghai Sixth People’s Hospital, Shanghai, China; 5 Department of Critical Care Medicine, Shanghai Fengxian District Central Hospital, Shanghai, China; 6 Department of Critical Care Medicine, Shanghai Eighth People’s Hospital, Shanghai, China; 7 Department of Critical Care Medicine, Shanghai Sixth People’s Hospital East Campus, Shanghai, China; 8 Department of Critical Care Medicine, Tongji University Affiliated Shanghai Tenth People’s Hospital, Shanghai, China; 9 Department of Critical Care Medicine, Shanghai Xuhui Central Hospital, Zhongshan-Xuhui Hospital, Fudan University, Shanghai, China

**Keywords:** Continuous glucose monitoring, Critically ill patients, Threshold of hyperglycaemia

## Abstract

**Aims/hypothesis:**

Continuous glucose monitoring (CGM) provides comprehensive information on the exposure to dysglycaemia. This study aimed to investigate the threshold of hyperglycaemia related to mortality risk in critically ill patients using CGM technology.

**Methods:**

A total of 293 adult critically ill patients admitted to intensive care units of five medical centres were prospectively included between May 2020 and November 2021. Participants wore intermittently scanned CGM for a median of 12.0 days. The relationships between different predefined time above ranges (TARs), with the thresholds of hyperglycaemia ranging from 7.8 to 13.9 mmol/l (140–250 mg/dl), and in-hospital mortality risk were assessed by multivariate Cox proportional regression analysis. Time in ranges (TIRs) of 3.9 mmol/l (70 mg/dl) to the predefined hyperglycaemic thresholds were also assessed.

**Results:**

Overall, 66 (22.5%) in-hospital deaths were identified. Only TARs with a threshold of 10.5 mmol/l (190 mg/dl) or above were significantly associated with the risk of in-hospital mortality, after adjustment for covariates. Furthermore, as the thresholds for TAR increased from 10.5 mmol/l to 13.9 mmol/l (190 mg/dl to 250 mg/dl), the hazards of in-hospital mortality increased incrementally with every 10% increase in TARs. Similar results were observed concerning the associations between TIRs with various upper thresholds and in-hospital mortality risk. For per absolute 10% decrease in TIR 3.9–10.5 mmol/l (70–190 mg/dl), the risk of in-hospital mortality was increased by 12.1% (HR 1.121 [95% CI 1.003, 1.253]).

**Conclusions/interpretation:**

A glucose level exceeding 10.5 mmol/l (190 mg/dl) was significantly associated with higher risk of in-hospital mortality in critically ill patients.

**Graphical Abstract:**

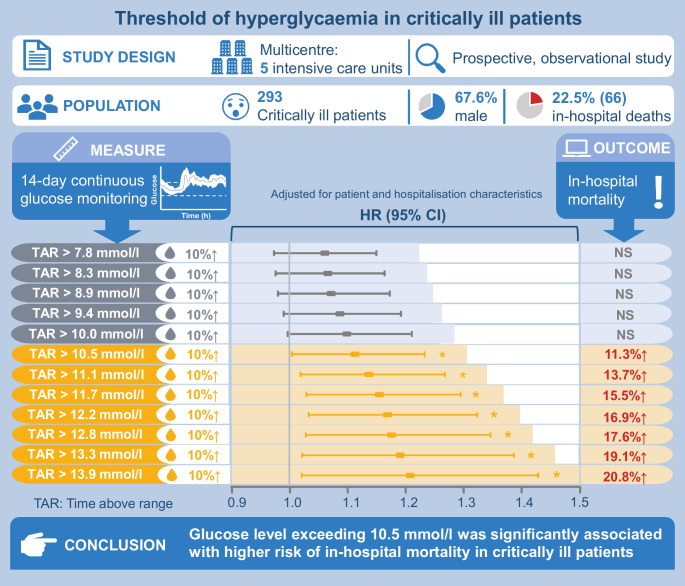

**Supplementary Information:**

The online version of this article (10.1007/s00125-024-06136-1) contains peer-reviewed but unedited supplementary material.


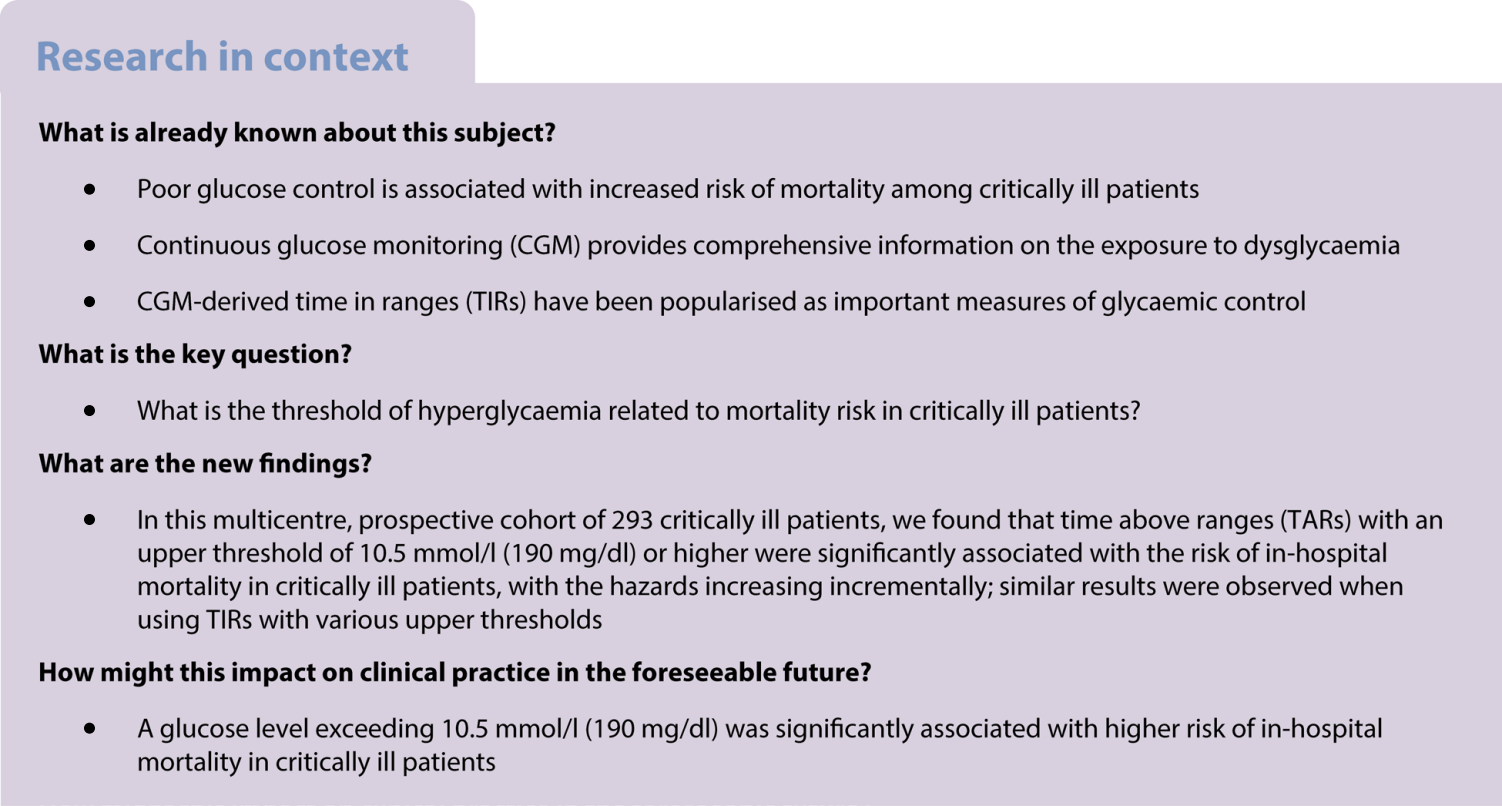


## Introduction

In critically ill patients, hyperglycaemia is common and is associated with poor outcomes [1, 2]. However, the results of the landmark Leuven study [3], which ushered in ‘tight glycaemic control’, were not reproduced in subsequent multicentre RCTs and meta-analyses [4–8]. Consequently, there is currently no firm consensus about how to manage blood glucose levels in critically ill patients.

Understanding the relationship between the glucose spectrum and clinical outcomes is fundamental for setting the glucose target. Compared with traditional blood glucose monitoring measurements, continuous glucose monitoring (CGM) provides a more complete glycaemic profile. Furthermore, the use of CGM in hospitals has been accelerated due to the COVID-19 pandemic [9–11]. Although the available evidence is limited, CGM has the potential to reduce the burden of nurses and may reduce hypoglycaemia incidence in critically ill patients [12–15]. Therefore, CGM provides new opportunities for more comprehensive and informative investigation of the association between hyperglycaemia and outcomes in critically ill patients.

Moreover, establishment of the core CGM metric is important for successful utilisation of CGM technology in routine clinical practice among critically ill patients. In recent years, the time in range (TIR), which refers to the percentage of time spent in an acceptable glucose range, has been popularised as an important measure for interpreting glucose data provided by CGM devices, with TIRs having been agreed upon by international guideline/consensus for use of diabetes management [16–18]. More recently, a consensus statement for CGM metrics in inpatient studies recommended TIRs as core outcome measures in the hospital setting [19]. With respect to the critically ill population, previous studies have observed a significant inverse association between time in range (TIR) and mortality risk, based on intermittent capillary, venous or arterial blood sampling data [20–22]. However, there is a lack of evidence linking TIR measured by CGM data to poor prognosis in critically ill patients. More importantly, the glucose range used to define TIR in this population remains to be explored [23, 24]. Therefore, based on CGM data, the current study examined the relationships between different predefined time above ranges (TARs)/TIRs using various glucose thresholds and the risk of in-hospital mortality in critically ill patients.

## Methods

### Study design and population

The INDIGO-ICU (INDices of contInuous Glucose monitoring and adverse Outcomes in Intensive Care Units) study is a multicentre, prospective and observational cohort study conducted in mixed medical/surgical intensive care units (ICUs) of five medical centres in Shanghai. It was designed to longitudinally examine the effects of quality of glucose control assessed by CGM on mortality risk in critically ill patients. The complete study protocol was approved by the Research Ethics Committees of Shanghai Sixth People’s Hospital Affiliated to Shanghai Jiao Tong University School of Medicine, and was in accordance with the Helsinki Declaration principles. Written informed consent was obtained from all participants.

Patients were consecutively recruited between May 2020 and November 2021. The following inclusion criteria were applied: (1) age ≥18 years; and (2) expected to stay in the ICU for at least 3 days. Exclusion criteria were as follows: (1) readmission to the ICU; (2) CGM data for fewer than 24 h; (3) receiving paracetamol (acetaminophen) >4 g/day or receiving high-dose ascorbic acid [10]; and (4) an admission diagnosis of hyperosmolar hyperglycaemic state or diabetic ketoacidosis. All participants, of Asian ethnicity, were drawn from various rural and urban regions throughout China (mostly from Shanghai). A total of 293 participants were included in the final analysis.

### Glucose control strategy

A standard ICU glucose control protocol was adopted. In accordance with ADA guidelines [16], the blood glucose target was 7.8–10.0 mmol/l (140–180 mg/dl) in our study. Blood glucose testing was performed using venous or capillary blood. The frequency of blood glucose monitoring ranged from hourly to every 4–6 h based on clinical need. Continuous i.v. administration of short-acting insulin (Tianmailin; Heifei Tianmai Biotechnology Development Co., China) using a micropump with a 1 U/ml concentration was initiated when blood glucose exceeded 10 mmol/l (180 mg/dl) for two successive readings. The initial insulin rates were recommended to be set using a sliding scale method, ranging from 2 to 6 U/h, according to the measured blood glucose levels (electronic supplementary material [ESM] Table 1). To avoid hypoglycaemia, we discontinued the i.v. infusion of insulin when blood glucose dropped below 7.8 mmol/l (140 mg/dl), and recommended i.v. administration of concentrated dextrose when blood glucose levels were below 3.9 mmol/l (70 mg/dl). For a single elevated blood glucose measurement, insulin was administered by s.c. injection (ESM Table 1).

Because CGM was not formally approved for hospital use, insulin adjustments in our study were guided via conventional blood glucose testing, and CGM data was blinded to the clinicians and nurses during the study period.

### CGM

We used FreeStyle Libre Pro Flash CGM (Abbott Diabetes Care, Alameda, CA, USA), a factory-calibrated sensor, for blinded s.c. interstitial glucose monitoring. The sensors were inserted on the first day of ICU admission, and then interstitial glucose levels were continuously measured every 15 min, generating a daily record of 96 glucose values for up to 14 days. TARs were calculated as the percentage of time above the glucose thresholds of 7.8, 8.3, 8.9, 9.4, 10.0, 10.5, 11.1, 11.7, 12.2, 12.8, 13.3 and 13.9 mmol/l (140, 150, 160, 170, 180, 190, 200, 210, 220, 230, 240 and 250 mg/dl) during the whole glucose monitoring period, denoted as TAR_>7.8_ to TAR_>13.9_. In addition, TIRs were calculated as the percentage of time in the glucose range between 3.9 mmol/l (70 mg/dl) and the same upper thresholds mentioned above during the same period, denoted as TIR_3.9–7.8_ to TIR_3.9–13.9_. Hypoglycaemia metrics including time below range (TBR) <3.9 mmol/l (70 mg/dl), TBR <3.0 mmol/l (54 mg/dl), AUC per day <3.9 mmol/l (70 mg/dl) and AUC per day <3.0 mmol/l (54 mg/dl) were calculated. Glycaemic variability metrics, including SD, CV and mean amplitude of glycaemic excursion (MAGE), were also calculated.

### Data extraction and outcome

The primary outcome was in-hospital mortality, defined as the occurrence of death during the hospital stay consecutive to the first ICU admission and prior to discharge. The following clinically relevant data was extracted from the hospital electronic medical record system and the ICU’s comprehensive database: demographic information, anthropometric measures and laboratory results. The sex of the study participants was determined through self-report during the initial demographic data collection. Disease severity was assessed by using the extracted Acute Physiology Score of the Acute Physiology and Chronic Health Evaluation II (APACHE II) [25].

### Statistical analysis

R version 4.0.3 (https://www.r-project.org) was used for the statistical analysis. Normality was tested by the Shapiro–Wilk Normality test first. Continuous variables were presented as mean ± SD or median (IQR, 25–75%) and categorical variables were presented as *n* (%). To compare the general characteristics of participants with or without outcome, *t* tests or Mann–Whitney *U* tests were conducted for normally or non-normally distributed continuous variables and χ^2^ tests were used for categorical variables. To investigate the upper threshold of glucose range, Cox proportional hazards regression was performed to assess the relationships of TAR_>7.8_ to TAR_>13.9_, with the risk of in-hospital mortality. Specifically, the values of TARs can be 0–100% and the HRs (95% CIs) were estimated by including different predefined TARs in the models separately as a continuous variable (per absolute 10% increase in TARs). In addition, the relationships of TIR_3.9–7.8_ to TIR_3.9–13.9_ with the risk of in-hospital mortality were also assessed, to further validate the threshold. Statistically significant covariates with a level of *p*<0.10, identified by univariate analysis and clinically relevant covariates based on prior literature (significant or not), were entered into the multivariate Cox regression model. The final model included age, sex, APACHE II score, the presence of diabetes, use of glucocorticoid in hospital and use of insulin in hospital. Then restricted cubic spline nested in the multivariate-adjusted Cox regression model was used to assess the dose–response relationship between levels of CGM metrics and in-hospital mortality. Moreover, explorative subgroup analyses in participants with and without pre-existing diabetes were performed. Two-tailed *p* values <0.05 were considered to indicate statistical significance.

## Results

A total of 293 critically ill patients (198 male, 95 female) were included in the final analysis. The clinical characteristics of the study population are presented in Table 1. Briefly, the mean ± SD age of the participants was 68±15 years and the mean ± SD APACHE II score was 19±6. Among them, 69 (23.5%) participants had pre-existing diabetes. During follow-up, 66 in-hospital deaths were identified (22.5%). Of those, there were 49 (21.9%) in-hospital deaths among participants without diabetes and 17 (24.6%) among those with diabetes (*p*=0.75). Participants who died before hospital discharge showed significantly higher APACHE II score and were less likely to receive glucocorticoid in hospital (both *p*<0.05), compared with those who survived to discharge.

**Table 1:** Clinical characteristics of the study population

Characteristic	Overall (*n*=293)	In-hospital mortality	
		No (*n*=227)	Yes (*n*=66)
Female, *n* (%)	95 (32.4)	70 (30.8)	25 (37.9)
Age, years	68±15	68±15	69±18
Systolic BP, mmHg	133±27	132±27	141±30
Diastolic BP, mmHg	76±17	75±16	78±20
Heart rate, beats/min	91±20	91±19	91±22
Respiratory rate, breaths/min	20 (17–22)	20 (17–22)	18 (16–20)
Diabetes, *n* (%)	69 (23.5)	52 (22.9)	17 (25.8)
Diagnostic category, *n* (%)			
Medical	65 (22.2)	55 (24.2)	10 (15.2)
Surgical	228 (77.8)	172 (75.8)	56 (84.8)
APACHE II score	19±6	18±6	22±6**
Mechanical ventilation, *n* (%)	249 (85.0)	188 (82.8)	61 (92.4)
Use of glucocorticoid in hospital, *n* (%)	53 (18.1)	47 (20.7)	6 (9.1)*
Use of insulin in hospital, *n* (%)	71 (24.2)	57 (25.1)	14 (21.2)
Dose of insulin, U/day^a^	37 (23–44)	38 (26–44)	30 (21–43)
Laboratory results			
Admission HbA_1c_, mmol/mol			
Patients with diabetes	65.7±24.6	66.0±26.1	64.6±20.1
Patients without diabetes	40.2±11.4	40.9±12.1	37.8±8.6
Admission HbA_1c_, %			
Patients with diabetes	8.2±2.3	8.2±2.4	8.1±1.8
Patients without diabetes	5.8±1.0	5.9±1.1	5.6±0.8
WBC, × 10^9^/l	12.5±6.5	12.5±6.3	12.5±7.0
ALT, U/l	26 (16–42)	26 (17–42)	25 (16–39)
AST, U/l	34 (22–55)	32 (22–52)	35 (24–56)
Creatine, µmol/l	82 (63–130)	82 (63–117)	84 (65–182)
K^+^, mmol/l	3.7±0.7	3.8±0.6	3.7±0.8
Na^+^, mmol/l	139.2±8.3	139.2±7.7	139.3±10.0
pH	7.4±0.2	7.4±0.2	7.4±0.1
HCT, %	32.4±8.2	32.6±8.3	31.9±7.8

Data are expressed as mean ± SD, median (IQR, 25 to 75%) or *n* (%)

^a^Doses of insulin were reported only for patients who used insulin in the hospital (*n*=71)

^*^p<0.05; **p<0.01

ALT, alanine aminotransferase; AST, aspartate aminotransferase; HCT, haematocrit; WBC, white blood cell count

**Fig. 1:**
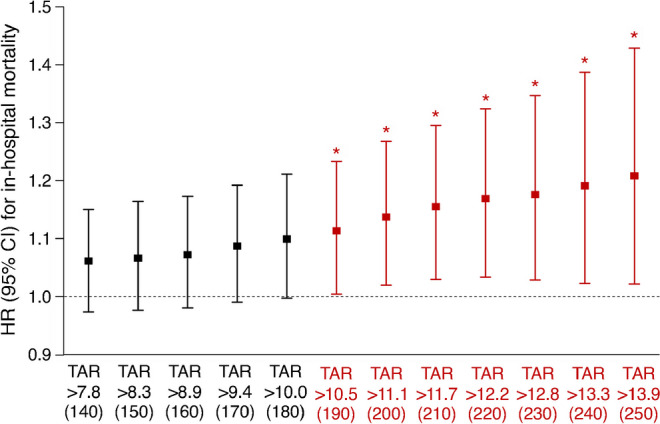
Forest plots illustrating HRs (95% CIs) for in-hospital mortality by each 10% increase in different CGM-derived TARs (%) defined using various glucose thresholds. *p<0.05, shown in red. The value of TARs can be 0–100% and the HRs (95% CIs) and *p* values were reported as per absolute 10% increment in TARs after adjustment for age, sex, APACHE II score, diabetes, use of glucocorticoid in hospital and use of insulin in hospital. The glucose thresholds shown on the *x*-axis are reported in units of both mmol/l and mg/dl (in parentheses)

Overall, participants wore the sensor for a median (IQR) period of 12.0 (7.0–14.0) days. After adjusting for age, sex, APACHE II score, diabetes, use of glucocorticoid in hospital and use of insulin in hospital, only TARs with a glucose threshold of 10.5 mmol/l (190 mg/dl) or higher (TAR_>10.5_ to TAR_>13.9_) were significantly associated with in-hospital mortality risk (Table 2, Fig. 1). As the thresholds for TARs glucose ranges increased from 10.5 mmol/l to 13.9 mmol/l (190 mg/dl to 250 mg/dl), the HR for in-hospital mortality increased incrementally with every 10% increase in TARs (Table 2, Fig. 1). Similarly, only TIRs with upper thresholds from 10.5 mmol/l to 12.2 mmol/l (190 mg/dl to 220 mg/dl) (TIR_3.9–10.5_ to TIR_3.9–13.9_) were significantly and negatively associated with the risk of in-hospital mortality, with the hazards of in-hospital mortality increasing incrementally (ESM Table 2).

**Table 2:** HRs for in-hospital mortality according to CGM-derived TARs defined using different thresholds

TAR threshold, %	HR	95% CI	*p* value
TAR >7.8 mmol/l (140 mg/dl)	1.061	0.973, 1.158	0.182
TAR >8.3 mmol/l (150 mg/dl)	1.066	0.976, 1.164	0.155
TAR >8.9 mmol/l (160 mg/dl)	1.072	0.980, 1.173	0.127
TAR >9.4 mmol/l (170 mg/dl)	1.087	0.990, 1.192	0.079
TAR >10.0 mmol/l (180 mg/dl)	1.099	0.997, 1.211	0.056
TAR >10.5 mmol/l (190 mg/dl)	1.113	1.004, 1.233	0.041
TAR >11.1 mmol/l (200 mg/dl)	1.137	1.019, 1.268	0.022
TAR >11.7 mmol/l (210 mg/dl)	1.155	1.029, 1.295	0.014
TAR >12.2 mmol/l (220 mg/dl)	1.169	1.033, 1.324	0.014
TAR >12.8 mmol/l (230 mg/dl)	1.176	1.028, 1.347	0.019
TAR >13.3 mmol/l (240 mg/dl)	1.191	1.022, 1.387	0.025
TAR >13.9 mmol/l (250 mg/dl)	1.208	1.021, 1.429	0.028

HRs and 95% CIs were calculated for each 10% increase in the TARs. Models were adjusted for age, sex, APACHE II score, diabetes, use of glucocorticoid in hospital and use of insulin in hospital

**Fig. 2:**
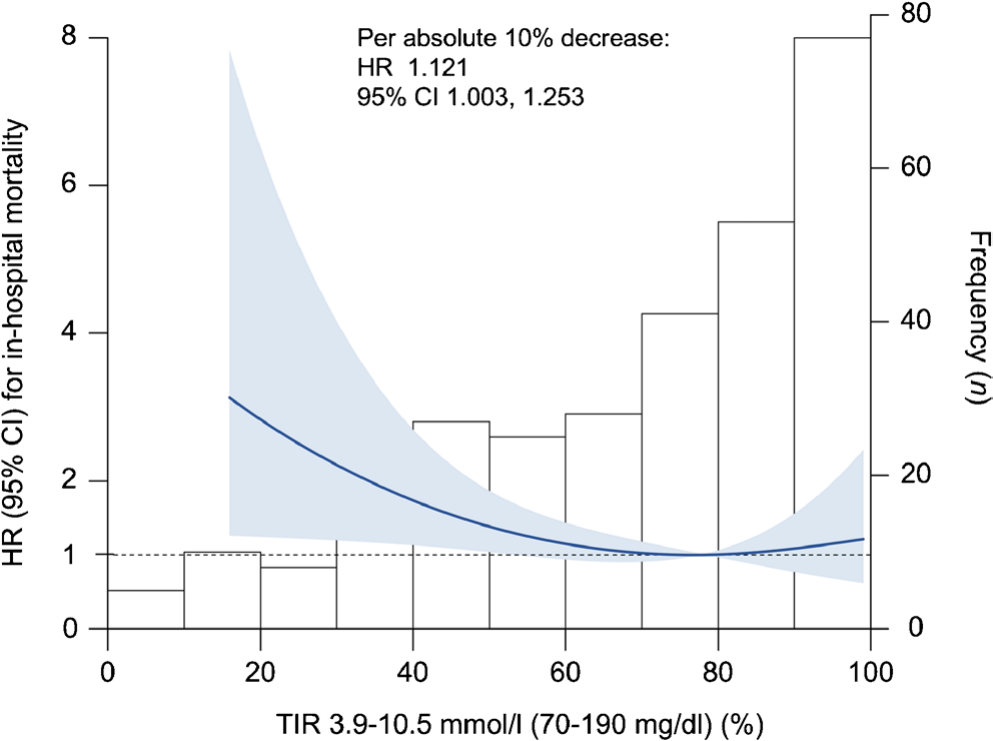
HRs (95% CIs) for in-hospital mortality by different levels of TIR (%) defined with a target range of 3.9–10.5 mmol/l (70–190 mg/dl) after adjustment for age, sex, APACHE II score, diabetes, use of glucocorticoid in hospital and use of insulin in hospital. The blue line represents the HR and the shaded blue area represents 95% CI. The dashed line indicates an HR of 1. Bars represent frequency (*n*)

Restricted cubic spline analysis suggested a significantly linear negative relationship between TIR_3.9–10.5_ and the risk of in-hospital mortality in critically ill patients (*p* for non-linear=0.193). Furthermore, restricted cubic spline curve showed that when TIR_3.9–10.5_ <52.7%, the risk of in-hospital mortality increases with decreasing TIR_3.9–10.5_ (Fig. 2). For per absolute 10% decrease in TIR_3.9–10.5_, the risk of in-hospital mortality was increased by 12.1% (HR 1.121 [95% CI 1.003, 1.253]) after adjustment for confounders (Fig. 2).

**Fig. 3:**
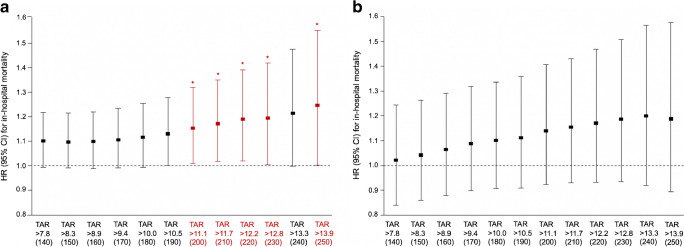
Forest plots illustrating HRs (95% CIs) for in-hospital mortality by different predefined TARs (%) among subpopulations stratified according to pre-existing diabetes status. (**a**) Participants without diabetes (*n*=224). (**b**) Participants with diabetes (*n*=69). *p<0.05, shown in red. The value of TARs can be 0–100% and the HRs (95% CIs) and *p* values were reported as per absolute 10% increment in TARs after adjustment for age, sex, APACHE II score, use of glucocorticoid in hospital and use of insulin in hospital. The glucose thresholds shown on the *x*-axis are reported in units of both mmol/l and mg/dl (in parentheses)

Subgroup analyses stratified by pre-existing diabetes status showed that in participants without diabetes, only TARs with a threshold of 11.1 mmol/l (200 mg/dl) or above (except at 13.3 mmol/l [240 mg/dl]) were significantly associated with the risk of in-hospital mortality after adjustment for covariates, with HRs for in-hospital mortality increasing incrementally (Fig. 3a and ESM Table 3). In participants with diabetes, however, there were no statistically significant associations between different predefined TARs and mortality risk (Fig. 3b and ESM Table 4).

The relationships of CGM-derived hypoglycaemia metrics and glycaemic variability metrics with the risk of in-hospital mortality are presented in ESM Table 5. We found that higher SD (1-SD: HR 1.316 [95% CI 1.024, 1.692]) and MAGE (1-SD: HR 1.331 [95% CI 1.024, 1.730]) were significantly associated with higher risk of in-hospital mortality. However, the relationships of CV and hypoglycaemia metrics with the in-hospital mortality risk did not reach statistical significance (all *p*>0.05).

## Discussion

In the current multicentre, prospective, observational study, we found that TARs with thresholds of 10.5 mmol/l (190 mg/dl) or higher were significantly related to the risk of in-hospital mortality in critically ill patients. The same threshold of hyperglycaemia associated with mortality risk was observed when considering TIRs with different upper thresholds. Therefore, the optimal upper glucose range for defining TIR in critically ill patients may be set near 10.5 mmol/l (190 mg/dl).

Understanding the relationship between the glucose spectrum and clinical outcomes is essential for setting the glucose target. The present results suggest that critically ill patients with sensor glucose level exceeding 10.5 mmol/l (190 mg/dl) may have an increased risk of mortality, similar to the findings of some previous RCTs targeting different blood glucose ranges [4–6]. For example, in the landmark NICE-SUGAR trial [4], critically ill patients assigned to intensive glycaemic management (4.4–6.1 mmol/l [80–110 mg/dl]) derived no significant treatment advantage compared with patients with more moderate glycaemic targets (7.8–10 mmol/l [140–180 mg/dl]) and had slightly but significantly higher mortality rate. Although debated, most professional societies currently recommend more ‘moderate’ glycaemic management for critically ill adults and suggest a blood glucose value of 10 mmol/l (180 mg/dl) or greater to trigger the use of insulin therapy [1, 16]. More recently, the large multicentre TGC-fast trial [26] showed that tight glucose control, in the context of delaying parenteral feeding to beyond the first week in ICU and the use of a performant algorithm, did not affect mortality and may protect liver and kidney function in critically ill patients, thus rekindling the long-debated question of glycaemic control in the ICU setting. Regarding this issue, our results seem to support a ‘moderate’ glycaemic management. However, it should be pointed out that the question of whether or not targeting a sensor glucose level <10.5 mmol/l (190 mg/dl) would reduce mortality risk remains to be addressed in future RCTs.

CGM has the potential to improve glucose control in the hospital setting, although core CGM metrics specific to inpatient care remains to be established [27]. Of the numerous metrics generated from CGM, TIRs have been recommended by international consensus statements and guidelines as important measures of glucose control [16, 17, 19]. With respect to the critically ill population, based on retrospective analysis of intermittent capillary, venous or arterial blood sampling data, TIR has been found to be negatively associated with the risk of mortality [20–22]. In addition, in a post hoc analysis of the SPRINT study data, TIR was observed to clearly discriminate the quality of glucose control between the SPRINT and Pre-SPRINT cohorts, despite similar median glucose values [28]. Compared with these previous studies, the main strength of the current study lies in the use of CGM data for the calculation of TIRs, which can provide more comprehensive information on the glucose profile throughout the day. Our results further provide evidence for the association between TIR/TAR measured by CGM data and mortality risk in critically ill patients. Moreover, our results indicate that 10.5 mmol/l (190 mg/dl) may be the appropriate upper limit of the ‘target’ glucose range used to define TIRs in critically ill patients. Taken together, TIRs, as valuable clinical measures, are worthy of more attention when using CGM technology and in the design of future interventional trials in critically ill patients. Of note, the ‘target’ glucose range used to define TIRs remains to be explored in different subpopulations based on clinical or participant-related outcomes [23, 24, 29, 30].

Besides, our results show that as the thresholds increase, every absolute 10% increase in TARs is related to incrementally higher risk of mortality. This could be explained by the effect of more ‘severe’ hyperglycaemia on mortality [31]. Compared with TIR, TAR is a more suitable metric for investigating the effect of hyperglycaemia, as it focuses on the relative exposure to hyperglycaemia, whereas TIR quantifies the relative exposure to ‘euglycaemia’ and hence reflects the risk of both hyperglycaemia and hypoglycaemia.

The main strengths of the current study include a prospective, multicentre study design and the use of 14 day CGM. However, there are also some limitations. First, it should be noted that the CGM device used in this study (FreeStyle Libre Pro) could be less accurate in the hypoglycaemia range [32], although this also applies to most other types of currently available CGM [33–35]. Therefore, the lower threshold of TIR, as well as the relationships between CGM-derived hypoglycaemia metrics and outcomes in critically ill patients, remains to be investigated in future studies, as new CGM technology with better performance in hypoglycaemia becomes available. Second, although we prospectively determined diabetes status at the onset of ICU admission based on all available information, the diabetes status adjusted in the model may not have been completely accurate. The potential influence of undiagnosed diabetes on our findings cannot be excluded. Third, due to the limited sample size of the subgroups stratified by diabetes status, the results of the subgroup analysis should be interpreted with caution. Future studies are warranted to further investigate the relationships between diabetes status, CGM metrics and outcomes in critically ill patients. Fourth, because of the limited scope of the medical records used in our study, the nutrition supply data was not available in this study.

In conclusion, we found that TARs/TIRs with an upper threshold of 10.5 mmol/l (190 mg/dl) or higher were significantly associated with the risk of in-hospital mortality in critically ill patients. These results suggest that critically ill patients with sensor glucose levels of 10.5 mmol/l (190 mg/dl) or higher may have an increased risk of mortality. In addition, based on our series of studies [29, 30], it is essential to acknowledge that when using TIR in clinical practice, different glucose ranges should be defined according to the characteristics of different populations, as well as treatment purpose. Future RCTs are warranted to determine whether targeting a sensor glucose level <10.5 mmol/l (190 mg/dl) would reduce the risk of mortality in critically ill patients.

### Supplementary Information

Below is the link to the electronic supplementary material.

Supplementary file1 (PDF 108 KB)

## Data Availability

Restrictions apply to the availability of data generated or analysed during this study to preserve patient confidentiality or because they were used under licence. Data are however available from the corresponding authors upon reasonable request.

## Abbreviations

APACHE II: Acute Physiology and Chronic Health Evaluation II
CGM: Continuous glucose monitoring
ICU: Intensive care unit
MAGE: Mean amplitude of glycaemic excursion
TAR: Time above range
TBR: Time below range
TIR: Time in range
