# Supplementary material for: Threshold of hyperglycaemia associated with mortality in critically ill patients: a multicentre, prospective, observational study using continuous glucose monitoring
Source: Diabetologia. 2024 Apr 3;67(7):1295–303. doi: 10.1007/s00125-024-06136-1 (PMC11153265; doi:10.1007/s00125-024-06136-1)
Supplement: Supplementary file 1 — Supplementary file1 (PDF 108 KB) [file 125_2024_6136_MOESM1_ESM.pdf]

Supplementary material

Table of Content

ESM table 1. Insulin regimen. 2

ESM table 2. Hazard ratios for in-hospital mortality according to CGM-derived TIRs defined using different thresholds. 3

ESM table 3. Hazard ratios (95% CIs) for in-hospital mortality by different CGM-derived TARs (%) defined using various glucose thresholds in patients without diabetes (n = 224).......... 4

ESM table 4. Hazard ratios (95% CIs) for in-hospital mortality by different CGM-derived TARs (%) defined using various glucose thresholds in patients with diabetes (n = 69).......... 5

ESM table 5. Hazard ratios for in-hospital mortality according to other CGM metrics.......... 6

ESM table 1. Insulin regimen.

1. Continuous intravenous (IV) insulin infusion regimen

**(1) Initiation:**	
Blood glucose exceeded 180 mg/dl on 2 successive readings.	
**(2) Rate of insulin infusion at initiation**	
Glucose (mg/dL[mmol/L]):	Rates (U/h):
• 181 ~ 220 (10.1 ~ 12.2)	2
• 221 ~ 260 (12.3 ~ 14.5)	3
• 261 ~ 300 (14.6 ~ 16.6)	4
• 301 ~ 350 (16.7 ~ 19.4)	5
• > 350 (19.4)	6, notify the clinicians

2. Subcutaneous insulin regimen: a single elevated blood glucose

Glucose (mg/dL[mmol/L]):	Doses (U):
• 181 ~ 220 (10.1 ~ 12.2)	4
• 221 ~ 260 (12.3 ~ 14.5)	6
• 261 ~ 300 (14.6 ~ 16.6)	8
• 301 ~ 350 (16.7 ~ 19.4)	10
• > 350 (19.4)	14, notify the clinicians

3. Hypoglycemia protocol

Glucose (mg/dL[mmol/L]):	Treatment:
• < 140 (7.8)	Discontinue insulin
• < 70 (3.9)	Discontinue insulin and administer a 10 ~ 15g concentrated IV dextrose; check blood glucose level after 30 minutes, and if it remains under 70 mg/dL (3.9 mmol/L), give dextrose again until the blood glucose being above 100 mg/dL (5.6 mmol/L).
• > 180 (10.0)	Restarting insulin infusion, and the initial insulin rate is halved

ESM table 2. Hazard ratios for in-hospital mortality according to CGM-derived TIRs defined using different thresholds.

TIRs defined using different thresholds, %	HRs	95% CIs	*p* values
TIR 3.9-7.8 mmol/l (70-140 mg/dl)	1.087	0.978, 1.209	0.124
TIR 3.9-8.3 mmol/l (70-150 mg/dl)	1.086	0.979, 1.205	0.120
TIR 3.9-8.9 mmol/l (70-160 mg/dl)	1.089	0.981, 1.209	0.108
TIR 3.9-9.4 mmol/l (70-170 mg/dl)	1.101	0.991, 1.224	0.072
TIR 3.9-10.0 mmol/l (70-180 mg/dl)	1.113	0.999, 1.239	0.052
TIR 3.9-10.5 mmol/l (70-190 mg/dl)	1.121	1.003, 1.253	0.045
TIR 3.9-11.1 mmol/l (70-200 mg/dl)	1.137	1.013, 1.276	0.030
TIR 3.9-11.7 mmol/l (70-210 mg/dl)	1.144	1.017, 1.287	0.026
TIR 3.9-12.2 mmol/l (70-220 mg/dl)	1.141	1.009, 1.290	0.035
TIR 3.9-12.8 mmol/l (70-230 mg/dl)	1.131	0.995, 1.286	0.059
TIR 3.9-13.3 mmol/l (70-240 mg/dl)	1.119	0.978, 1.280	0.103
TIR 3.9-13.9 mmol/l (70-250 mg/dl)	1.111	0.968, 1.276	0.135

HRs and 95% CIs were calculated for each 10% decrease in TIRs. Models were adjusted for age, sex, APACHE II score, diabetes, use of glucocorticoid in hospital and use of insulin in hospital.

Abbreviation: CGM: continuous glucose monitoring; TIR, time in range; HR, hazard ratios; CI, confidence interval.

ESM table 3. Hazard ratios (95% CIs) for in-hospital mortality by different CGM-derived TARs (%) defined using various glucose thresholds in patients without diabetes (*n* = 224).

TARs defined using different thresholds, %	HRs	95% CIs	*p* values
TAR >7.8 mmol/l (140 mg/dl)	1.101	0.995, 1.217	0.062
TAR >8.3 mmol/l (150 mg/dl)	1.098	0.992, 1.216	0.071
TAR >8.9 mmol/l (160 mg/dl)	1.099	0.990, 1.221	0.077
TAR >9.4 mmol/l (170 mg/dl)	1.106	0.991, 1.235	0.071
TAR >10.0 mmol/l (180 mg/dl)	1.117	0.994, 1.254	0.063
TAR >10.5 mmol/l (190 mg/dl)	1.131	1.000, 1.279	0.050
TAR >11.1 mmol/l (200 mg/dl)	1.154	1.010, 1.319	0.035
TAR >11.7 mmol/l (210 mg/dl)	1.172	1.018, 1.350	0.027
TAR >12.2 mmol/l (220 mg/dl)	1.191	1.020, 1.391	0.027
TAR >12.8 mmol/l (230 mg/dl)	1.195	1.005, 1.420	0.044
TAR >13.3 mmol/l (240 mg/dl)	1.214	0.998, 1.476	0.052
TAR >13.9 mmol/l (250 mg/dl)	1.247	1.003, 1.552	0.047

HRs and 95% CIs were calculated for each 10% increase in TARs. Models were adjusted for age, sex, APACHE II score, use of glucocorticoid in hospital and use of insulin in hospital.

Abbreviation: CGM: continuous glucose monitoring; TAR, time above range; HR, hazard ratios; CI, confidence interval.

ESM table 4. Hazard ratios (95% CIs) for in-hospital mortality by different CGM-derived TARs (%) defined using various glucose thresholds in patients with diabetes (*n* = 69).

TARs defined using different thresholds, %	HRs	95% CIs	*p* values
TAR >7.8 mmol/l (140 mg/dl)	1.022	0.841, 1.243	0.824
TAR >8.3 mmol/l (150 mg/dl)	1.042	0.860, 1.263	0.672
TAR >8.9 mmol/l (160 mg/dl)	1.065	0.879, 1.290	0.519
TAR >9.4 mmol/l (170 mg/dl)	1.088	0.897, 1.319	0.392
TAR >10.0 mmol/l (180 mg/dl)	1.101	0.907, 1.336	0.332
TAR >10.5 mmol/l (190 mg/dl)	1.112	0.909, 1.360	0.303
TAR >11.1 mmol/l (200 mg/dl)	1.139	0.923, 1.406	0.224
TAR >11.7 mmol/l (210 mg/dl)	1.154	0.930, 1.431	0.193
TAR >12.2 mmol/l (220 mg/dl)	1.171	0.933, 1.470	0.173
TAR >12.8 mmol/l (230 mg/dl)	1.187	0.934, 1.508	0.161
TAR >13.3 mmol/l (240 mg/dl)	1.200	0.919, 1.566	0.180
TAR >13.9 mmol/l (250 mg/dl)	1.188	0.894, 1.577	0.235

HRs and 95% CIs were calculated for each 10% increase in TARs. Models were adjusted for age, sex, APACHE II score, use of glucocorticoid in hospital and use of insulin in hospital.

Abbreviation: CGM: continuous glucose monitoring; TAR, time above range; HR, hazard ratios; CI, confidence interval.

ESM table 5. Hazard ratios for in-hospital mortality according to other CGM metrics.

CGM metrics	HRs	95% CIs	*p* values
Mean sensor glucose, mmol/L	1.317	1.001-1.731	0.049
TBR < 70 mg/dl (3.9 mmol/L), %	0.994	0.773-1.278	0.964
TBR < 54 mg/dl (3.0 mmol/L), %	1.047	0.829-1.322	0.700
AUC <3.9 mmol/L, mmol/l × min	0.952	0.752-1.205	0.683
AUC <3 mmol/L, mmol/l × min	0.979	0.789-1.215	0.847
Standard deviation, mmol/L	1.316	1.024-1.692	0.032
Coefficient of variation, %	1.131	0.868-1.474	0.360
MAGE, mmol/L	1.331	1.024-1.730	0.032

HRs and 95% CIs were calculated for each 1-SD increase in CGM metrics. Models were adjusted for age, sex, APACHE II score, diabetes, use of glucocorticoid in hospital and use of insulin in hospital.

Abbreviation: CGM: continuous glucose monitoring; HR, hazard ratios; CI, confidence interval; TBR, time below range; AUC, area under curve; MAGE, mean amplitude of glycemic excursion.
